# N-acetylcysteine-loaded electrospun mats improve wound healing in mice and human fibroblast proliferation *in vitro*: a potential application of nanotechnology in wound care

**DOI:** 10.22038/ijbms.2020.41550.11078

**Published:** 2020-12

**Authors:** Ramin Seyedian, Elham Shabankareh Fard, Maryam Najafiasl, Majid Assadi, Sasan Zaeri

**Affiliations:** 1 Department of Pharmacology, School of Medicine, Bushehr University of Medical Sciences, Bushehr, Iran; 2 Department of Environmental Health Engineering, Faculty of Health, Bushehr University of Medical Sciences, Bushehr, Iran; 3 Department of Chemical Engineering, School of Petroleum, Gas, and Petrochemical Engineering, Persian Gulf University, Bushehr, Iran; 4 Nuclear Medicine and Molecular Imaging Research Center, Bushehr University of Medical Sciences, Bushehr, Iran

**Keywords:** Electrospun nanofiber, In vitro, Mouse, N-acetylcysteine (NAC), Oxidative stress, Wound

## Abstract

**Objective(s):**

N-acetylcysteine (NAC) has gained attention recently in dermatology as a unique anti-oxidant. In light of progress in nanotechnological methods, it was hypothesized that loading NAC onto nanofibers would positively affect skin wound healing. The objective of this study was to fabricate NAC-loaded electrospun mats and test their effect on wound healing* in vivo* and *in vitro*.

**Materials and Methods:**

Polyvinyl alcohol (PVA)-based mats loaded with NAC at three concentrations were electrospun and characterized in terms of physicochemical properties and drug release profile. Human fibroblast cells (*in vitro*) and mouse full-thickness skin wounds (*in vivo*) were treated with mats for 5 and 14 days, respectively. Wound area, tissue histopathology, fibroblast proliferation and cellular oxidative state were evaluated.

**Results:**

Mats containing 5% PVA/NAC showed thinner fibers with suitable physicochemical properties and a sustained drug release profile. PVA/NAC (5%) mats enhanced fibroblast proliferation and attachment *in vitro*. The mats resulted in significant wound closure with high levels of re-epithelialization and collagen fiber synthesis on day 14 post-surgery *in vivo.* The mats also reduced granulation tissue and edematous stroma to a higher extent. These findings were accompanied by a significant decrease in tissue lipid peroxidation and higher superoxide dismutase activity, which may explain how NAC improved wound healing.

**Conclusion:**

We propose an NAC-loaded nanofibrous mat that takes the advantage of a porous nanoscaffold structure to release NAC in a sustained manner. This mat may be a promising candidate for further clinical evaluation.

## Introduction

Skin injuries, burns, diabetic ulcers and other insults to the intact skin are considerable challenges in dermatology and skin care. The selection of appropriate wound-healing agents or dressings plays a pivotal role in skin tissue restoration, prevention of dermal or systemic infections, and ultimately in the aesthetic outcome of wound healing. An alternative therapeutic approach to conventional topical wound-healing formulations such as gels, creams, ointments etc. is the use of drug-loaded engineered scaffolds as skin wound dressings (1).

 Drug-loaded wound dressings with a nanofibrous structure possess a number of key characteristics required for good wound healing, such as resemblance to the extracellular matrix, attachment to dermal fibroblasts and high healing efficiency, anti-microbial properties and high surface-to-volume ratio, wettability, and porosity. Electrospinning polymer solutions are widely utilized to fabricate nanofibers. In this connection, a variety of polymers of natural (e.g. gelatin, chitosan, and alginate) or synthetic origin (e.g. polyvinyl alcohol and polyethylene oxide) have been used for this purpose (2). 

The process of wound healing is complex and involves several successive but overlapping phases, including hemostasis, inflammation, proliferation and maturation (remodeling). Indeed, coordinated actions among all agents in the wound healing process are required for complete skin regeneration with minimal side effects such as infections or scar formation (3). The reduction/oxidation (redox) status of the wounded tissue is a major determinant among the many cellular and subcellular factors that influence the wound healing process. Reactive oxygen species (ROS) can be considered as second messengers acting directly or indirectly on the cells involved in wound healing (4). Although oxidative stress may be partly beneficial in wound healing, excess oxidative stress can disturb the normal wound repair process by inhibiting major biological activities required for appropriate skin regeneration (5). At the molecular level, gene expression of matrix metaloproteinases (MMP) and proinflammatory cytokines may be increased by ROS. In contrast, excess ROS production can directly destroy extracellular matrix proteins or indirectly affect them by inducing proteolysis. Moreover, ROS may impair the actions of skin fibroblasts and keratinocytes (6). Accordingly, searching for agents with anti-oxidant properties may lead to the discovery of promising wound-healing candidates for skin wound care. 

N-acetylcysteine (NAC) is a thiol-containing compound with significant anti-oxidant activity that has a number of clinical applications (7). This compound can either directly scavenge free radicals or indirectly counteract them by taking part in the production of glutathione, a major cellular anti-oxidant molecule (8). Accordingly, oral and parenteral NAC was suggested as a promising agent for wound healing in some experimental and clinical studies (9, 10). In light of the known anti-oxidant activity of NAC on one hand, and the advantages of applying nanotechnology in designing nanofibrous mats as wound dressings on the other hand, the objective of the present study was to fabricate, characterize and evaluate the wound healing efficacy of NAC-loaded electrospun nanofibers *in vitro* and *in vivo*. 

## Materials and Methods

***Materials***

NAC, polyvinyl alcohol (PVA) (MW 72,000 Da) and phosphate-buffered saline (PBS) tablets were supplied by Merck Co. (Germany). Glutaraldehyde 25% (v/v) was purchased from Fluka Co. (UK). Ketamine and xylazine vials were supplied by Alfasan Co. (the Netherlands). Dulbecco’s modified Eagle’s medium (DMEM) and fetal bovine serum (FBS) were purchased from Gibco Co. (Germany). Penicillin/streptomycin and DMSO were purchased from Sigma Co. (USA), and the cell viability reagent MTT (3-(4, 5-dimethyl-2-thiazolyl)-2,5-diphenyl-2H-tetrazolium bromide) was from BioFroxx Co. (Germany). Double distilled water (dd H_2_O) was used as the solvent to fabricate mats.

***Preparation of polymer solutions and electrospun mats***

The PVA concentration and electrospinning parameters were adopted from previous work with some modifications (11). In brief, PVA solution at a concentration of 6% (w/v) was prepared by dissolving PVA powder in hot water (80 °C) with stirring for 3 hrs. After the PVA solution had cooled, NAC powder was added to the solution to obtain a final concentrations of 2.5 or 5% (w/v). NAC-loaded PVA solutions were stirred for 6 hrs until complete dissolution. To fabricate nanofibrous mats, a mono-jet electrospinning apparatus manufactured by Fanavaran Nano Meghyas Co. (Iran) was used. A 5-ml syringe attached to a blunt-head needle (18 G) was used to inject polymer solutions. The distance from the needle tip to the collector was set at 12 cm with an applied voltage of 15-17 kV. Injection rate was adjusted to 0.25 ml/hr, and the electrospun fibers were collected onto aluminum foil on a rotating drum (120 rpm). The electrospun fibers were subsequently dried under a vacuum for 6 hrs and carefully peeled off the foil. Electrospun fibers fabricated from PVA 6% (w/v) solution containing 0%, 2.5% and 5% NAC (w/v) were designated hereafter neat PVA, PVA/NAC 2.5% and PVA/NAC 5% mats, respectively. To cross-link mats, they were exposed to 10% (v/v) glutaraldehyde solution vapor in an enclosed desiccator dish for 12 hrs at room temperature. The mats were subsequently washed with dd H_2_O to remove excess unreacted glutaraldehyde. 

***Characterization of the electrospun mats***

Size and morphology of the electrospun mats were evaluated with scanning electron microscopy (SEM) (CamScan-MV2300, US). A thin layer of the electrospun mat (1 × 1 cm) was coated with gold, and an acceleration voltage of 20 kV was used to obtain SEM micrographs. The images were then imported into ImageJ Software v. 1.47 (National Institutes of Health, USA) to determine fiber diameters. Mean fiber diameter in each mat sample was calculated by measuring the diameter of at least 100 random fibers in that sample.

***Swelling and weight loss tests***

To investigate swelling, mats were immersed in PBS buffer (pH 7.4) at room temperature for 1 hr. The degree of swelling was determined based the following equation:

where *M* is the weight of each sample after immersion in the PBS buffer solution and *M*_i_ is the initial weight of that sample in the dry state. In addition, weight loss of the mats as a measure of their structural resistance against aqueous media was determined. Small pieces of mats (~50 mg) were accurately weighed initially and then immersed in 20 ml of PBS (pH 7.4) at 37 °C for 24, 48, 72 or 96 hrs. At each time point, the mat remnants were removed from the buffer solution, washed gently with dd H_2_O, and desiccated under vacuum to a final constant weight. Equation 2 was used to calculate the extent of weight loss (%) for each mat sample at the specified times:

where *M*_i_ is the initial weight of each sample (dry weight before immersion in PBS) and *M*_t_ is the sample weight at time *t* (dry weight after immersion in PBS).

***Porosity test***

Porosity of the mats was calculated by the following equation:

where *ρ*_m_ is the apparent calculated density and *ρ*_0_ is the density of the polymer in its bulk form [*ρ*_0__ (PVA)_ = 1.3 g/cm^3^]. In order to calculate apparent density (*ρ*_m_), the mats were cut into 2 × 2 cm squares, stacked together to yield an average thickness of about 200 µm according to a digital caliper, and then weighed in a digital precision scale. Finally, the mass-to-volume ratio of each mat sample (*ρ*_m_) was calculated for use in the porosity equation.

***Fourier-transform infrared spectroscopy (FTIR)***

The chemical structure of the electrospun PVA-based mat with and without NAC was characterized with FTIR spectroscopy (Perkin Elmer, Frontier, USA). The electrospun mats were powdered and mixed with KBr at 1:100 (w/w). A scanning range of 400–4000 cm^-1^ with 4 cm resolution was used to record the spectra.

***Drug release profile and kinetic modeling***

To determine NAC release behavior from the electrospun mats, NAC-loaded mats (25 mg) were immersed in 25 ml PBS buffer (pH = 7.4, 37 °C) under constant gentle shaking. After 1, 2, 3, 6, 12, 24, 72 and 96 hr, 2 ml of the solution was sampled for NAC spectrophotometric measurement at 193 nm according to a calibration curve in a range of 0 to 70 µg/ml NAC. The missing volume was replaced with fresh PBS buffer solution (2 ml) so that total volume remained constant. Cumulative drug release (%) was calculated based on the following equation:

where C_t_ is the NAC concentration at the specified time, and C_∞_ denotes the NAC maximum final concentration at plateau.

To further investigate the release mechanism, the release data were fitted into a number of known release kinetic models, i.e. zero order, first order, Higuchi and Korsmeyer–Peppas models (equations 5 to 8) (12). The last model is well-established for describing drug release kinetics from polymer matrices (13).

where k_0_, k_1_, k_H_ and k_P_ are the release rate constants, *n* is the diffusion exponent for the release mechanism in the Korsmeyer–Peppas model. C_t _and C_∞_ were defined previously. 

***In vitro*** **cell proliferation assay**

The MTT assay was used to assess the *in vitro* effects of NAC-loaded nanofibers on cell proliferation and biocompatibility in cultured human foreskin fibroblasts (HNFF-P18). Briefly, HNFF-P18 cells (Pasteur Institute Cell Bank, Iran) were cultured in DMEM supplemented with 10% (v/v) FBS, penicillin (100 U/ml) and streptomycin (100 μg/ml) in a 5% CO_2_ incubator at 37 °C. Cells from the third and fourth passages were seeded onto DMEM-soaked nanofibers in a 24-well multi-plate at a density of 2.5 × 10^4^ cells per well and incubated for 1, 3 and 5 days. Sterile MTT solution (5 mg/ml) was added to each well, and then the cell-seeded nanofibers were incubated at 37 °C for 4 hrs. Subsequently, absorbance was read with a microplate reader (Power waveX, Bio-tek Instruments, USA) at 570 nm. The proliferation rate (%) of fibroblasts was calculated with the following formula in which A_s_, A_c_ and A_b_ denote absorbance of the sample, control and blank, respectively. Each experiment was repeated four times.

***In vivo skin wound healing***

*Animals and wound model*

The inbred male albino mice (25–30 g) used for the wound healing experiment were purchased from the experimental animal breeding center of Bushehr University of Medical Sciences (BPUMS), Iran. They were transported to the experimental lab 1 week before the onset of experiments, to allow for familiarization. All mice had free access to water and food pellets. The temperature was kept at 24 °C, with a 12-hr light-dark cycle. All animals were handled in accordance with guidelines for animal care and with the approval of the Ethics Committee of BPUMS. 

The mice were initially anesthetized by intraperitoneal injection of a mixture of ketamine/xylazine (60/10 mg/kg). The interscapular region was chosen for wound creation because this region was inaccessible to the animals with their paws or mouth. The area was shaved and disinfected with ethanol 70%, and a circular excisional full-thickness wound of 1 cm in diameter was made with sharp, curved-tip surgical scissors. Care was taken to excise the skin smoothly and not to damage the layers beneath the skin.

*Experimental design*

A total of 40 mice were randomly and equally distributed in four experimental groups. Topical treatments were as follow: Normal saline (group 1), NAC 5% (w/v) in PVA gel (group 2), neat PVA mat [without NAC] (group 3), or PVA/NAC 5% mat (group 4). An NAC gel formulation condition (treatment group 2) was intentionally included so that comparisons could be made between nanofibers and the gel formulation of NAC. The gel was made up of PVA 6% (w/v) solution, which contained NAC at a final concentration of 5% (w/v). Because PVA solution behaves rheologically more like gels than aqueous fluids, we used the term PVA gel. To reduce microbial contamination, all topical preparations were sterilized under ultraviolet-C light for 30 min before application on the wounds. After the appropriate treatment was applied, the wounds were bandaged with simple sterile gauze. All animals were treated at 24-hr intervals. Half of the animals in each group received treatment for 7 days, while the other half received treatment until day 14. To determine changes in wound area, the wounds were photographed with a digital camera immediately after wound creation (day 0) and on days 3, 7, and 14 post-surgery. During photography, a ruler was placed near the wound for scaling purpose. On days 7 or 14, the wound tissue plus a small portion of the surrounding intact skin was excised. Half of the sample was stored in 10% formalin for histopathological analysis, and the other half was stored at ­80 °C for biochemical assay. After the wound biopsies were obtained, the animals were killed by intracardial injection of a high dose of ketamine/xylazine. 

*Macroscopic (wound area) measurements*

Photographs of wounds were processed with ImageJ Software v. 1.47 to calculate the wound areas. For each skin wound, the area calculated on different days post-surgery was normalized to its area on day 0. This compensated for any variations in the size of the initial wound. Hence, macroscopic data were reported as area (%) on days 3, 7 or 14 post-surgery as calculated by the following equation:

where *A*_d_ is wound area on the selected day and *A*_0_ is wound area on day 0 (the day of surgery). 

*Microscopic (histopathologic) measurements*

Hematoxylin-eosin (H and E) and Masson’s trichrome stains were used to evaluate the histopathological changes in wound tissues on day 7 and 14 post-surgery. The tissue preparation and staining process are described briefly as follow: cross-sections were obtained of the excised wounds to include both wound tissue and the neighboring intact skin. Samples were fixed in 3.7% formalin, embedded in paraffin, cut into 5-µm sections, and stained in accordance with routine protocols for H and E and Masson’s trichrome staining. The histopathological parameters recorded in this study were re-epithelialization, neovascularization, collagen density, granulation tissue, and the levels of fibroblasts and polymorphonuclear (PMN) leukocytes. These parameters were scored as 0 (absent), 1 (mild), 2 (moderate), or 3 (severe) based on a semiquantitative scaling system proposed in a previous study (14) with some modifications (Table 1).

*Anti-oxidant activity assay*

Skin wound tissues were homogenized in ice-cold PBS buffer (pH=7.4) and centrifuged at 3500 rpm at 4 °C for 20 min. The supernatants were then separated for anti-oxidant assay. The Bradford method was used to determine total protein levels with bovine serum albumin as the standard protein (15). The redox status in the wounded tissues was assessed by measuring the level of lipid peroxidation and superoxide dismutase (SOD) activity. Lipid peroxidation was determined based on the thiobarbituric acid reactive substances (TBARS) method, in which the levels of malondialdehyde (MDA) were measured at 532 nm and reported in nmol/mg protein. The SOD activity assay was based on the conversion of superoxide anions to hydrogen peroxide and oxygen molecules by SOD, and activity in sample tissues was reported as units/mg protein. These assays were performed according to the instructions provided by the manufacturers of the MDA and SOD assay commercial kits (Abnova, Taiwan).

***Statistical analysis***

All data from all experiments and assays were obtained from at least triplicate measurements and were reported as the mean±standard deviation except where noted in the text. The Kruskal–Wallis test followed by pairwise comparisons was used to analyze the microscopic (histopathological) data. The macroscopic (wound area) and other data were analyzed with one-way ANOVA and the Tukey’s *post hoc* test. All differences at *P*<0.05 were considered statistically significant. All data analyses were performed with SPSS^®^ Software version 21.

## Results

***Fiber size and distribution***

**Figure 1:**
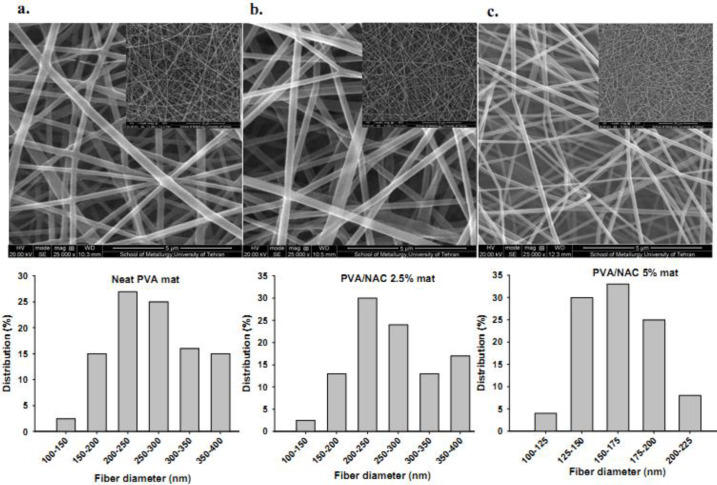
Morphology and the corresponding size distribution of nanofibers in neat PVA, PVA/NAC 2.5% and PVA/NAC 5% mats. Scale bars represent 5 µm in the large SEM photos. Inset photos at lower magnifications are also provided for each mat type. NAC: N-acetylcysteine; PVA: Polyvinyl alcohol

SEM photomicrographs of electrospun fibers with different NAC concentrations are shown in Figure 1 together with the corresponding size distribution graphs. Non-woven, beadless and uniform nanofibers were observed at different NAC concentrations. Fiber thickness was significantly influenced by the presence of NAC. Mean fiber diameter in the PVA/NAC 5% mat was 26.3% lower than in PVA/NAC 2.5% mats and 29.6% lower than in neat PVA mats (F=5.23, df=2, *P=*0.024) (Table 2).

***Swelling and weight loss***

**Figure 2:**
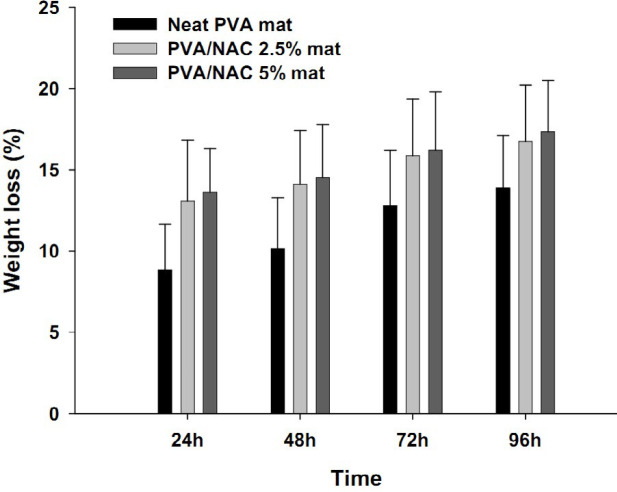
Weight loss (gravimetric assay) in different PVA-based electrospun mats in PBS buffer solution (pH 7.4, 37 °C) at different time intervals

Percentage swelling rates in the electrospun mats are presented in Table 2. The degree of swelling in NAC 5% mats was slightly higher than that in NAC 2.5% and neat PVA mats. However, the differences were not statistically significant (F=0.83, df=2, *P=*0.16). Weight loss assays were conducted to assess structural resistance of the electrospun scaffolds against the surrounding medium. Figure 2 shows the trends in weight loss in all three mats (with and without NAC) at different time intervals upon incubation in PBS buffer. Weight loss in all mats increased slightly with time. Although weight loss in the PVA/NAC 2.5% and 5% mats appeared to be greater than that in the neat PVA mat, but the differences were not statistically significant (F=1.09, df=2, *P=*0.18).

***Porosity of the mats***

Porosity values were high, a finding that favors cellular accommodation and attachment (Table 2). Porosity in the PVA/NAC 5% mat was 11.0% higher than the neat PVA mat and 4.4% higher than the PVA/NAC 2.5% mat. The higher porosity of the PVA/NAC 5% mat may be related to its thinner fiber diameter.

***FTIR analysis***

**Figure 3:**
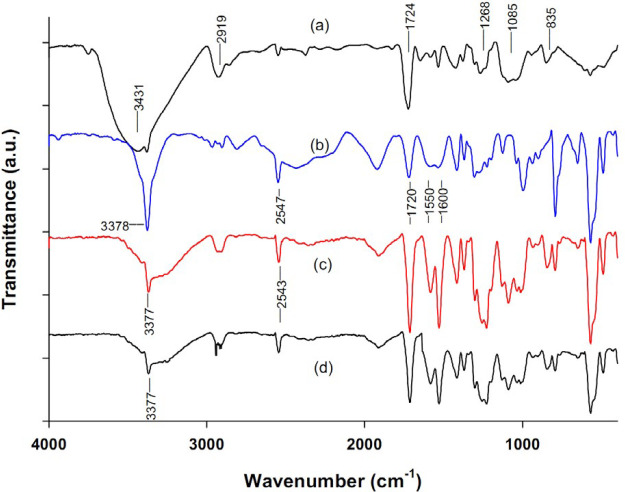
FTIR spectra of (a) pure PVA powder, (b) pure NAC powder, and the PVA/NAC electrospun mat (c) before and (d) after cross-linking with glutaraldehyde vapor. The main diagnostic peaks are marked in each spectrum

NAC: N-acetylcysteine; PVA: Polyvinyl alcohol; FFTIR: Fourier-transform infrared spectroscopy

**Figure 4:**
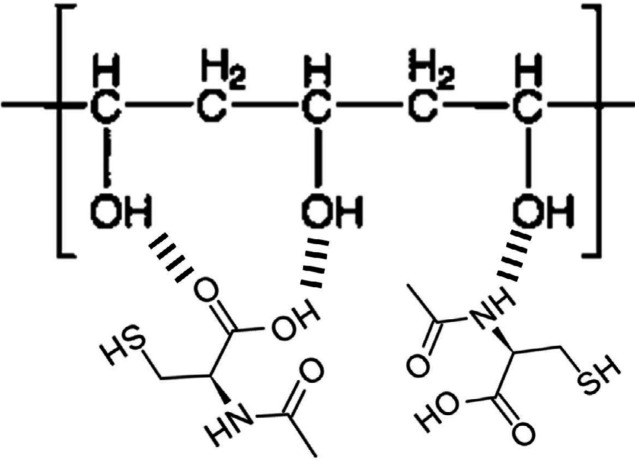
Schematic presentation of possible hydrogen-bonding interactions between PVA and NAC molecules in the electrospun nanofiber mats. NAC: N-acetylcysteine, PVA: Polyvinyl alcohol

The FTIR spectra of pure PVA and NAC powders as well as PVA/NAC electrospun mats are shown in Figure 3. For the PVA/NAC mat, the spectra of both cross-linked and non-cross-linked fibers are shown. For the PVA spectrum (Figure 3a), the absorption peaks at 1268, 1085 and 835 cm^-1^ can be attributed to C-H bending, C-O stretching and C-C stretching vibrations, respectively (16). Stretching vibration of the residual acetyl (C-C=O) group in PVA produced a sharp peak at 1724 cm^-1^. The broad peak at 3431 cm^-1^ was due to stretching of the hydroxyl group (-OH) in PVA. The peak at 2919 cm^-1^ corresponded to asymmetric bending of C-H group. Figure (3b) shows the characteristic FTIR profile of NAC, in which peaks at 3378, 2547 and 1720 cm^-1^ corresponded to the stretching vibrations of N-H, S-H and C=O groups in the NAC molecule, respectively (17). Adjacent peaks at 1550 and 1600 cm^-1^ represented the carboxyl group (COO^-^) in NAC (18). FTIR spectra for PVA/NAC mats before cross-linking with glutaraldehyde (Figure 3c) revealed two distinctive peaks at 3377, and 2543 cm^-1^, which corresponded well with the aforementioned peaks at 3378 (N-H) and 2547 (S-H) cm^-1^ for NAC. Other peaks (e.g. 1550 and 1600 cm^-1^) for NAC were also present in the PVA/NAC mat spectrum. The cross-linked PVA/NAC fibers (Figure 3d) demonstrated a similar spectrum although intensity of the –OH peak (3431 cm^-1^) reduced compared to non-cross-linked PVA/NAC fibers. This reflected the reaction of glutaraldehyde with some of the hydroxyl groups in PVA (19). Notably, NAC remained mainly unreacted after cross-linking, since the diagnostic NAC peaks at 3377, 2543 and 1720 cm^-1^ were still present (Figure 3d). Hence, the NAC present in the electrospun mats did not undergo major, significant interactions with PVA molecules before or after cross-linking. Of note, the observed changes in the peak shapes and wavenumbers in the PVA/NAC mat spectrum (before cross-linking) compared to the spectra for each pure agent can be explained by hydrogen bonds between electronegative (oxygen and nitrogen) and hydrogen atoms in the agents. Figure 4 shows the proposed hydrogen bonding between the NAC and PVA molecules.

***NAC release assay***

**Figure 5:**
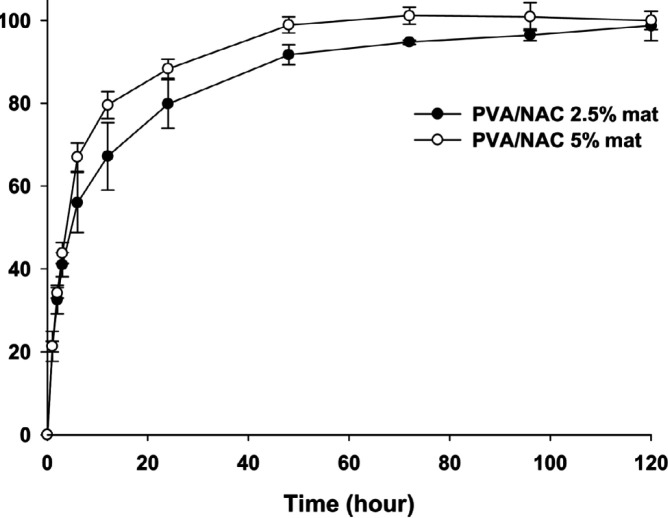
Cumulative drug release (%) profiles of PVA/NAC 2.5% and 5% mats in PBS buffer solution (pH 7.4, 37 °C). NAC concentration was measured at 1, 2, 3, 6, 12, 24, 48, 72, 96 and 120 hrs (n =3)

NAC: N-acetylcysteine; PVA: Polyvinyl alcohol; PBS: phosphate-buffered saline

Drug release profiles of PVA-based mats loaded with NAC 2.5% and 5% for a period of 120 hrs are depicted in Figure 5. The release of NAC from all mats was relatively fast, with approximately 80% of the drug being released within the first 24 hr. This was followed by a sustained release rate for the rest of the observation period. Mats containing both 2.5% and 5% NAC showed almost identical release profiles although the PVA/NAC 5% mat exhibited slightly faster release. The final concentration of NAC released was 22.2±0.7 µg/ml for PVA/NAC 2.5% mats and 45.9±2.3 µg/ml for PVA/NAC 5% mats.

Table 3 presents the results of fitting release data to different mathematical kinetics models. Except for the zero-order model, other models showed convincing fits in terms of regression coefficients, with the highest coefficient in the Korsmeyer–Peppas model (R^2^=0.97). Values of n (diffusion exponent) in this model were 0.46 and 0.54 for PVA/NAC 2.5% and 5% mats, respectively. Because these values lie within the range of 0.45 < n < 1, they imply a non-Fickian release mechanism in which release is governed by both drug diffusion and polymer swelling (20).

***Biocompatibility characterization of nanofiber scaffolds by MTT assay and SEM***

**Figure 6:**
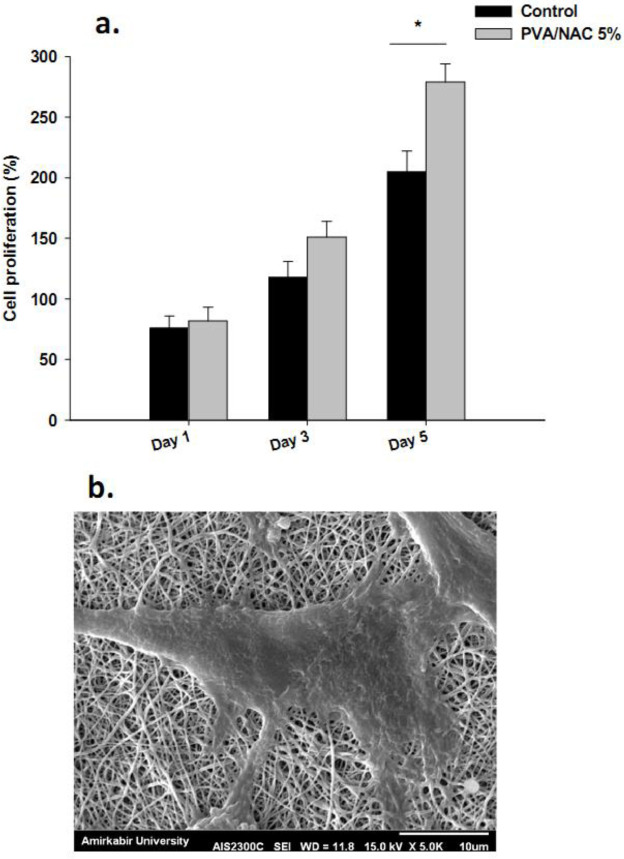
(a) Proliferation rate (%) of human foreskin fibroblasts incubated with PVA/NAC 5% mats for 1, 3 and 5 days. Bars represent the mean±standard deviation (n=4/group). (b) Representative SEM photograph of a fibroblast showing considerable stretching and adhesion to the nanofiber scaffold after 5 days of incubation with the PVA/NAC 5% mat

NAC: N-acetylcysteine; PVA: Polyvinyl alcohol

Figure 6 a shows the percentage cell proliferation rate on days 1, 3 and 5 of incubation with the PVA/NAC 5% mat. This mat formulation enhanced the proliferation of human foreskin fibroblasts to a significantly higher extent than in the control group (cells without the scaffold) on day 5 of incubation (*P* < 0.001). Figure 6b shows a SEM image of activated fibroblasts on the PVA/NAC 5% scaffold. Fibroblasts were fully extended and formed pseudopods, which facilitated interactions with the drug-eluting nanofibers. The SEM image also shows appropriate morphology of the fibroblasts and confirms the results of MTT assay. 

***Macroscopic (wound area) analyses***

**Figure 7:**
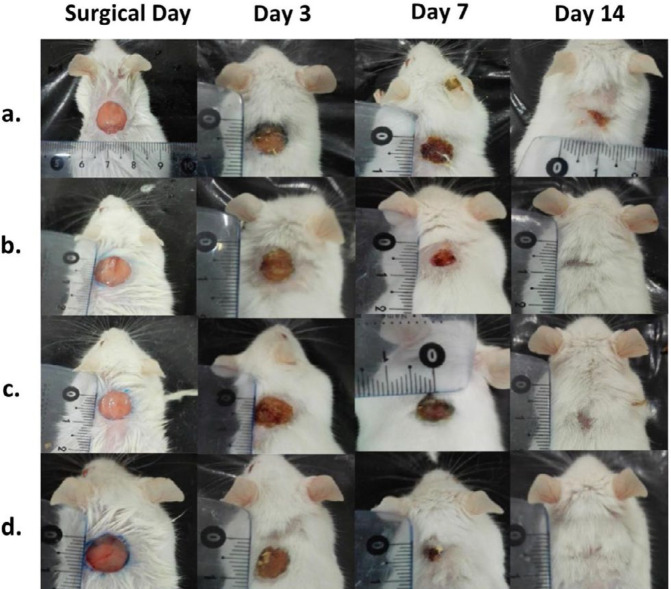
Representative photos of full-thickness skin wounds treated with (a) normal saline, (b) NAC 5% in PVA gel, (c) neat PVA mats, or (d) PVA/NAC 5% mats on days 0, 3, 7 and 14 post-surgery

NAC: N-acetylcysteine; PVA: Polyvinyl alcohol

Figure 7 shows representative photos of wounds after treatment for different post-surgical periods with normal saline, NAC 5% gel, neat PVA or PVA/NAC 5% mats. In addition, the corresponding means wound areas are reported in Table 4. The largest wound area was observed in the normal saline group on day 3 post-surgery (86.7%±4.7), and the smallest area was observed in wounds treated with a PVA/NAC 5% mat on day 14 post-surgery (14.1%±3.0). As expected, wounds in all experimental groups had smaller areas on day 14 than on days 3 or 7 post-surgery. One-way ANOVA disclosed significant differences in mean of wound areas between the experimental groups on day 7 (*F*=8.89, df= 3, *P=*0.0003) and day 14 post-surgery (*F*=4.50, df = 3, *P=*0.012). *Post hoc* analysis showed that PVA/NAC 5% mats, on day 7, resulted in significantly smaller wound sizes than did normal saline (20%, *P=*0.0003) or neat PVA mats (15.8%, *P=*0.007). On day 14 post-surgery, PVA/NAC 5% mats led to further reductions in wound size compared to normal saline (35%, *P=*0.012) or neat PVA mats (31.5%, *P=*0.037). Of note, treatment with NAC 5% gel yielded significantly better results than normal saline on day 7 (*P=*0.036) but not on day 14 post-surgery (*P=*0.770).

***Microscopic (histopathologic) analyses***

**Figure 8:**
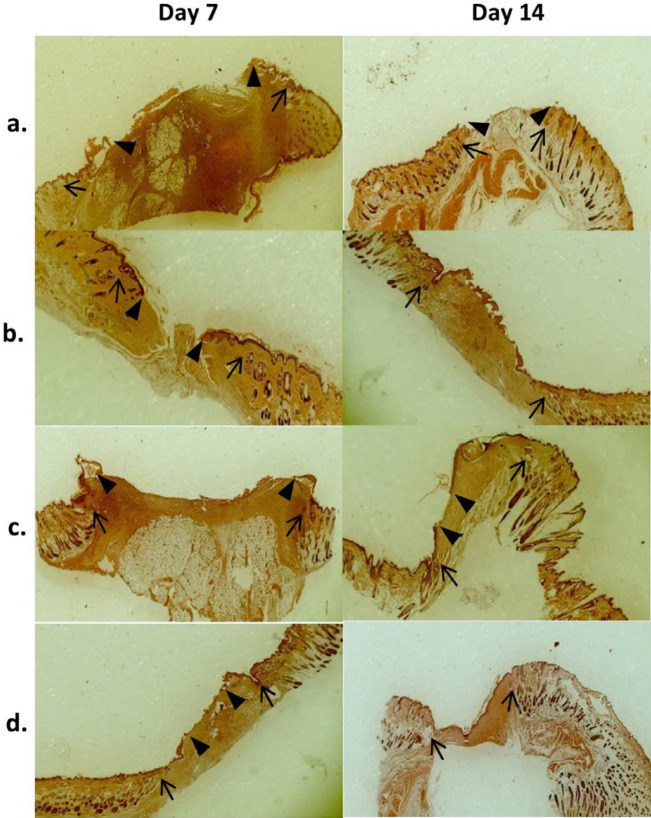
Representative low magnification (×40) photomicrographs of H and E-stained wound tissues treated with (a) normal saline, (b) NAC 5% in PVA gel, (c) neat PVA mat, or (d) PVA/NAC 5% mat after 7 or 14 days of treatment. Arrows (→) show wound margins and arrowheads () show re-epithelialization fronts. Wound stroma beneath the re-epithelialized area lacked skin adnexa in all experimental groups on day 14 post-surgery

NAC: N-acetylcysteine; PVA: Polyvinyl alcohol

**Figure 9:**
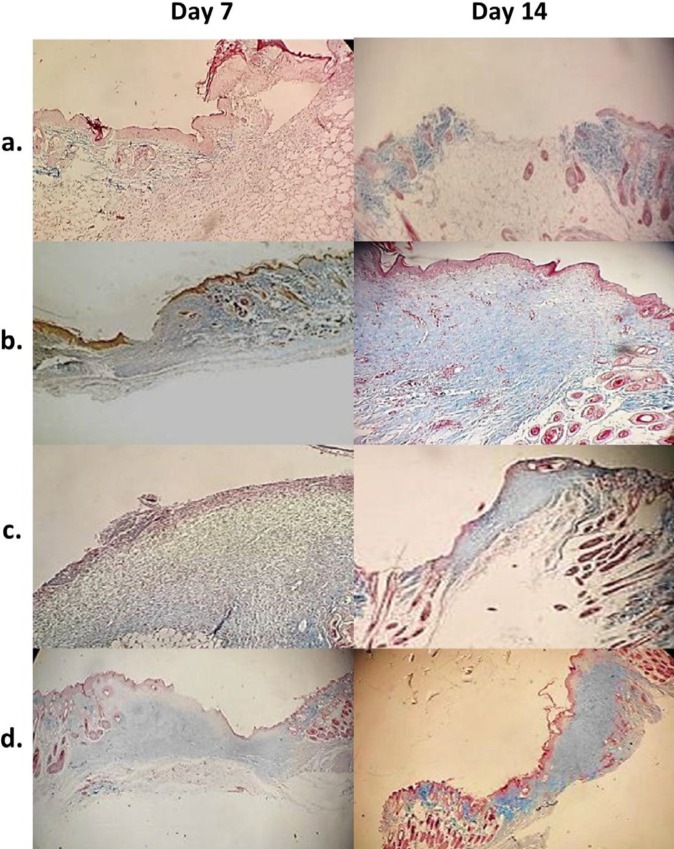
Representative low-magnification (×40-100) photomicrographs of Masson’s trichrome-stained wound tissues treated with (a) normal saline, (b) NAC 5% gel, (c) neat PVA mat, or (d) PVA/NAC 5% mat 7 or 14 days post-surgery. Collagen fibers were stained blue. The presence of collagen fibers was greatest in wounds treated with PVA/NAC 5% mats on day 14 post-surgery

NAC: N-acetylcysteine; PVA: Polyvinyl alcohol

Representative photomicrographs of H and E-stained wound tissues obtained on days 7 or 14 post-surgery are shown in Figure 8, and the corresponding wound tissues stained with Masson’s trichrome are presented in Figure 9. Seven days after wound creation in normal saline group, highly edematous granulation tissue was observed with high levels of PMNs and new vessels with thin endothelia (neovascularization) and low levels of re-epithelialization and fibroblasts (Figure 8a). Collagen production was negligible (Figure 9a). Partial improvements in wound healing were observed on day 14, but the healing process was still far from complete. Wounds treated with neat PVA mat showed relatively better wound healing profiles compared to normal saline on days 7 and 14 post-surgery. This was evident from the smaller amounts of granulation tissue and greater re-epithelialization on days 7 and 14 (Figure 8c) and especially from the higher collagen density on day 14 (Figure 9c). Histopathological findings in wounds treated with NAC 5% gel indicated relatively enhanced healing features compared to the normal saline group, which include lower levels of PMNs, new vessel formation and granulation tissue observed with re-epithelialization at a moderate (day 7) and high level (day 14) (Figure 8b). Collagen fiber production was also improved on days 7 and 14 (Figure 9b). In wounds treated with PVA/NAC 5% mats, re-epithelialization was more extensive than in the other groups on day 7, and was nearly complete on day 14 post-surgery. Notably, wounds were covered with a keratin layer on day 14. Minimal levels of PMNs, granulation tissue and neovascularization were found, and PMNs were partially replaced by macrophages and lymphocytes (Figure 8d). In addition, collagen fibers were prominent throughout the wound bed, with high levels of fibroblast proliferation (Figure 9d). Although wounds treated with PVA/NAC 5% mats showed a better healing profile than other treatments, the development of skin appendages did not recover even after 14 days of topical treatment.

**Figure 10:**
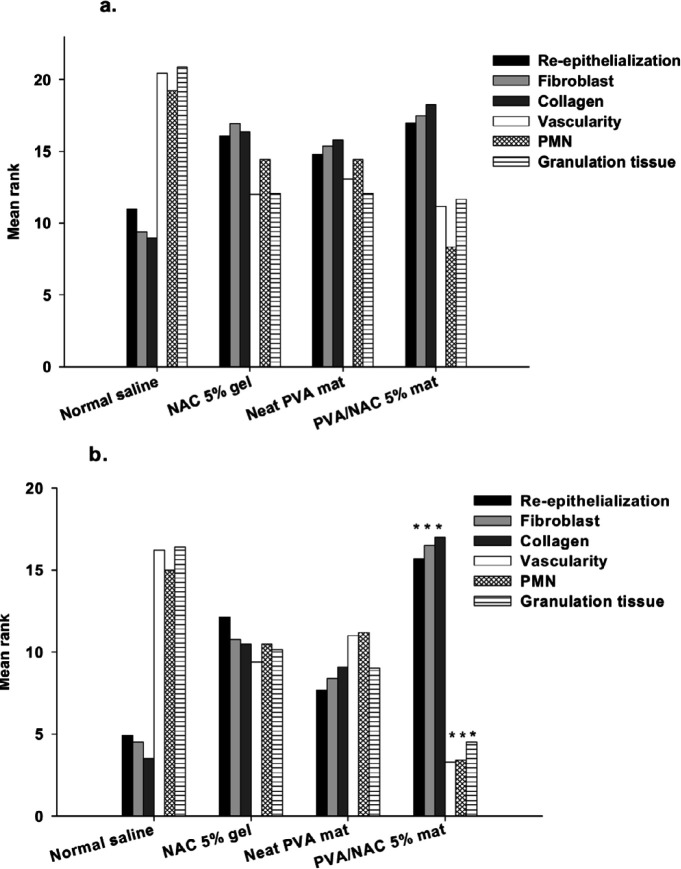
Mean ranks of different histopathological parameters involved in wound healing after topical treatment of the wounds with normal saline, NAC 5% gel, neat PVA, or PVA/NAC 5% mats for (a) 7 or (b) 14 days. The data were analyzed with the Kruskal–Wallis test followed by pairwise comparisons between groups (n=4–5/group)

The results of semiquantitative analyses of the histopathological parameters were in accordance with the above descriptive findings. For the data from day 7, statistical analysis failed to detect significant differences among experimental groups in re-epithelialization (χ^2^=2.94, df=3, *P*=0.40), fibroblast growth (χ^2^=26.35, df=3, *P*=0.09), collagen density (χ^2^=6.49, df=3, *P*=0.09), PMNs (χ^2^=6.93, df=3, *P*=0.07), neovascularization (χ^2^ =7.32, df=3, *P*=0.07), or granulation tissue (χ^2^=8.45, df=3, *P*=0.03) (Figure 10a). However, the data for day 14 post-surgery yielded significant differences among experimental groups in re-epithelialization (χ^2^=12.12, df=3, *P*=0.007), fibroblast growth (χ^2^=13.57, df=3, *P*=0.004), collagen density (χ^2^=16.59, df=3, *P*=0.001), PMNs (χ^2^=12.76, df=3, *P*=0.005), neovascularization (χ^2^=15.25, df=3, *P*=0.002), and granulation tissue (χ^2^=13.13, df=3, *P*=0.004) (Figure 10b). Although mean ranks of the parameters in NAC 5% gel and neat PVA mats showed better wound healing compared to normal saline, but the differences were not statistically significant. In contrast, mean ranks of re-epithelialization, fibroblast growth and collagen density in wounds treated with PVA/NAC 5% mats were significantly higher than wounds treated with normal saline (*P*=0.007, 0.002 and <001, respectively). In addition, in wounds treated with PVA/NAC 5% mats the parameters associated with wound inflammation were lower than wounds treated with normal saline, e.g. PMNs (*P*=0.003), neovascularization (*P*=0.001) and granulation tissue (*P*=0.002).

**MDA and SOD assays**

**Figure 11:**
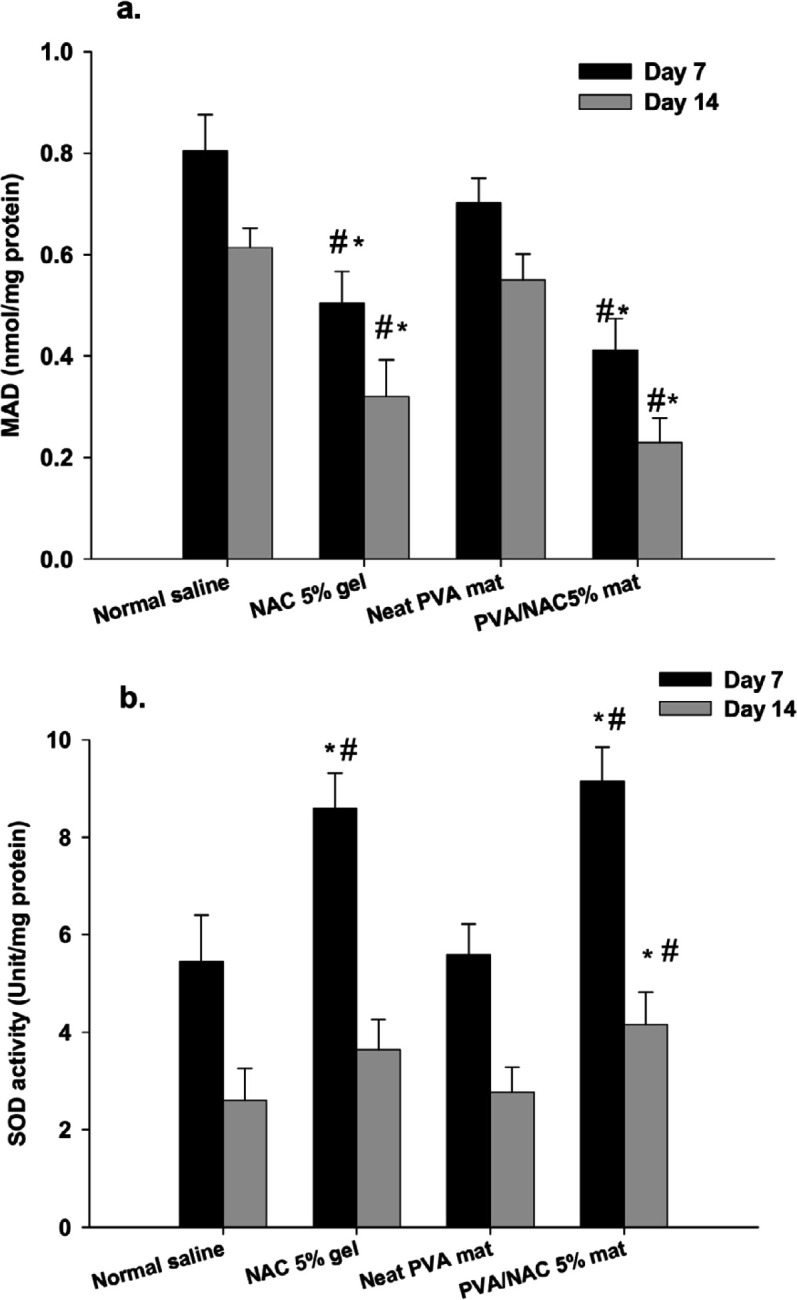
(a) MDA level and (b) SOD activity in wound tissues after treatment with normal saline, NAC 5% gel, neat PVA, or PVA/NAC 5% mats on day 7 or 14 post-surgery. The data were analyzed with one-way ANOVA followed by the Tukey’s post hoc test. Bars represent the mean±standard deviation (n= 4–5/group). * and # indicate significant differences (*P*<0.05) compared to normal saline and neat PVA mats, respectively

MDA: Malondialdehyde; SOD: Superoxide dismutase; NAC: N-acetylcysteine; PVA: Polyvinyl alcohol

Lipid peroxidation (MDA level) in wound tissues was significantly affected after 7 (*F*=39.80, df=3, *P*<0.001) and 14 (*F*=55.09, df=3, *P*<0.001) days of treatment (Figure 11a). Treatment with PVA/NAC 5% mats for 7 days significantly reduced MDA levels by 48% compared to normal saline, and by 41% compared to neat PVA mats (both *P*<0.001). Similarly, NAC 5% gel significantly decreased MDA levels by 37% (*P*<0.001) compared to normal saline, and by 28% (*P*=0.001) compared to neat PVA mats. On day 14 post-surgery, lipid peroxidation was generally lower in all experimental groups than on day 7. Again, compared to treatment with normal saline, MDA levels were lower in wounds treated with (67%, *P*<0.001) and NAC 5% gel (47%, *P*<0.001). Compared to wounds treated with neat PVA mats, MDA levels were 58% lower in the PVA/NAC 5% mat group (*P*<0.001) and 41% lower in the NAC 5% gel group (*P*<0.001).

SOD activity differed significantly among experimental groups on day 7 post-surgery (*F*=32.61, df=3, *P*<0.001) (Figure 11b). Compared to treatment with normal saline, SOD activity was 68% higher in wounds treated with PVA/NAC 5% mats (*P*<0.001) and 59% higher after treatment with NAC 5% gel (*P*<0.001). Compared to treatment with neat PVA mats, SOD activity was 66% higher in the PVA/NAC 5% group and 56% higher in the NAC 5% group (both *P*<0.001). All experimental groups had lower levels of SOD activity on day 14 than on day 7 post-surgery. However, the differences among groups remained statistically significant on day 14 post-surgery (*F*=6.60, df=3, *P*<0.001). The highest levels of SOD activity were observed in wounds treated with PVA/NAC 5% mats, and these values were 57% higher than those in wounds treated with normal saline (*P*=0.007) and 52% higher than wounds after treatment with PVA mats (*P*=0.027).

## Discussion

The skin is the largest organ in the body, which plays a major role in maintaining homeostasis. Wound healing after physical, chemical and microbial insults is a key process in the recovery and regeneration of lost skin tissues. Effective wound healing is a multiphase process that is affected by a number of key determinants, especially the redox state of the wounded tissue. Because the wound healing process can be enhanced or hindered by a variety of factors (21), finding new methods and strategies to enhance wound healing is of great concern for researchers and clinicians.

The present study investigated the role of NAC, a potent anti-oxidant agent, in nanofiber mats tested *in vivo* and *in vitro* for their ability to favor wound healing. The main innovation in the present work is the use of nanotechnology to fabricate NAC-eluting nanofibrous mats, in order to take advantage of the high surface-to-volume ratio, hydrophilicity and porosity of the mats to facilitate wound healing. A major finding was the ability of PVA-based fibrous mat containing NAC 5% to accelerate wound closure, enhance fibroblastic proliferation, and affect cellular and molecular processes in ways that favored enhanced wound healing. These effects can be attributed mainly to the suitable physicochemical properties of the fibers, and to the high capacity of NAC as a promising bioactive agent in the field of wound care.

Mats produced with PVA/NAC 5% generally exhibited a number of physicochemical properties required for effective wound dressings. PVA was chosen for electrospinning as a nontoxic polymer with high electrospinnability, which made it possible to blend NAC into PVA to fabricate electrospun nanofibers. The addition of NAC to the PVA solution not only did not interfere with electrospinnability of the polymer, but in fact reduced fiber diameter as the concentration of NAC increased. This may be due to the increase in polarity and conductivity of the drug-containing solution (22). Surface-to-volume ratio is another significant variable, which is directly proportional to fiber size (23). Because PVA/NAC 5% mats demonstrated lower average fiber diameters, they can be assumed to have a higher surface-to-volume ratio. This would be an advantage in the wound healing process (24).

Porosity is another major parameter, which plays role in the fabrication of nanofiber mats for tissue regeneration and wound healing purposes. If a wound dressing mat is highly porous, more oxygen diffusion occurs in the wounded tissue and more fibroblasts can be accommodated between nanofibers, leading to the acceleration of wound healing (25). According to the findings for porosity, the higher NAC concentration (5% w/v) yielded higher porosity, which may be associated in part with the narrower fibers obtained with this NAC concentration.

The degree of swelling (hydrophilicity) and ability of a wound dressing mat to preserve water affects its effectiveness in wound healing. Because we found no significant difference in swelling between different mat formulations, we can attribute the similarity in swelling to the fact that all three mat types were composed of PVA. However, swelling also depends on other parameters such as porosity, surface-to-volume ratio of the fibers, and the nature of the added drug (26, 27). Given that NAC is a hydrophilic chemical (28), we expected swelling to increase together with the concentration of NAC in fibers, as was observed in PVA/NAC 5% mates compared to neat PVA mats. This finding can be attributed to hydrophilic interaction between NAC on the fiber surface and the aqueous medium. Although this effect of NAC was not strong enough to cause significant differences in swelling rates between the different 3 mats, the ability of PVA/NAC 5% mats to retain more water may help prevent wound dryness and nutrient loss, as well as promoting cellular proliferation and growth on the mat (29, 30).

A simple gravimetric assay was used to evaluate structural endurance of the electrospun fibers against PBS buffer at different time intervals. An important feature of any wound dressing mat is resistance to rapid disintegration when applied on the wound (31). Weight loss in fibers is closely associated with swelling, because swelling can lead to more rapid disintegration. In mats containing higher NAC levels, weight loss was slightly but nonsignificantly greater than neat PVA mat. The reason for this slightly higher weight loss in PVA/NAC 5% mats may be the same as for the swelling results discussed above. As an aqueous medium, PBS buffer makes full contact with hydrophilic surfaces and penetrates more easily into porous structures (32).

It is routine to evaluate drug effects on cell lines before tissues or the whole body in order to document how the targeted cells respond. We therefore investigated the biocompatibility of the nanofiber scaffolds with the MTT assay and SEM in the human foreskin fibroblastic cell line before implanting the nanofiber mats in animals. PVA/NAC 5% mats were nontoxic to the cells, and in fact promoted cell proliferation and attachment, an effect that can be attributed to the ability of NAC to promote cell proliferation (33). In line with our findings, Zhu *et al*. fabricated a NAC-containing nanoscaffold and showed that it had a positive effect on the viability of rat bone marrow-derived stroma cells (rBMSCs) (34).

*In vivo* wound healing experiments with our electrospun mats were carried out in full-thickness excisional wounds in mice for a period of 14 days. Throughout the experimental period, care was taken to maintain wound cleanliness, and no wound infections were observed. In addition to the PVA/NAC 5% mat, a simple NAC 5% gel formulation was included to study the effects of NAC alone in the absence of the nanofiber scaffold. Although wound closure was more evident in wounds treated with the NAC 5% gel and PVA/NAC 5% mat compared to other treatments on day 3, statistically significant wound closure was only observed on day 7 and later. In line with this finding, Oguz *et al*. showed that NAC 3% cream resulted in significant wound closure in rats, and that the effect was time-dependent (35). Of note, the smallest wound area was obtained with the PVA/NAC 5% mat, which can be attributed in part to its specific surface properties that maintain wound moisture at an appropriate level and thereby favor wound closure. 

Analysis of the histopathological (microscopic) changes is another means of evaluating the wound healing process, which provides deeper information of the process and complements the macroscopic findings. Favorable histopathological parameters (more re-epithelialization, fibroblast proliferation and collagen synthesis, less inflammation and granulation tissue) were more pronounced in tissues treated with NAC 5% gel and especially with PVA/NAC 5% mats. These findings correspond well with our macroscopic results. In line with these findings, it was reported that cell proliferation, collagenous expression of MMP-1 and re-epithelialization improved significantly after treatment with topical NAC *in vitro* and *in vivo* (36). In addition, Demir *et al*. showed that the oral or intraperitoneal administration of NAC to irradiated rats yielded better wound healing in terms of re-epithelialization, perianastomotic wound fibrosis, and other histopathological parameters (37). In addition, the administration of NAC promoted healing in ulcers caused by bullous morphea in a clinical study (38).

NAC provides cysteine (a thiol-bearing amino acid), which is involved in glutathione replenishment. It also possesses anti-oxidant and anti-inflammatory properties (39). In the wound healing process, inflammatory cells, fibroblasts and other cells produce ROS, which at high levels may disturb normal wound repair by damaging proteins, lipid membranes and DNA (5). Hence, the administration of NAC would be expected to modulate the redox state of wound tissues in favor of facilitating wound healing. In the present study, the anti-oxidant efficacy of topical treatments was investigated by measuring SOD activity and MDA levels in wound tissues to search for possible mechanistic explanations for the macroscopic and microscopic findings. As expected, NAC in the simple gel formulation or as a component in nanofibrous mats enhanced SOD activity (an important endogenous anti-oxidant enzyme) and reduced the breakdown products of lipid peroxidation (MDA levels) in wound tissues. Endogenous anti-oxidant enzymes such SOD, catalase and others promote wound repair by scavenging ROS (40). Although both NAC gel and PVA/NAC 5% mats promoted SOD activity on day 7 post-surgery, the effect was more pronounced latter, especially on day 14 post-surgery. The anti-oxidant effect of treatments was further evaluated by measuring MDA levels as a simple marker of the extent of oxidative damage in wound tissues. NAC-containing formulations produced lower MDA levels on day 7 and, to a greater extent, on day 14 post-surgery. In accordance with our findings, Aktunc *et al*. reported that the intraperitoneal administration of NAC (150 mg/kg) reduced MDA levels in wound tissues of both diabetic and nondiabetic mice compared to controls after 5 days of treatment (10). In addition, it was shown that NAC accelerated anastomotic wound healing, while decreasing serum MDA levels and increasing SOD activity in irradiated rats (37). Therefore, the present findings regarding the anti-oxidant activity of NAC in early and late phases of healing link the observed accelerated wound repair with the elevated activity of endogenous anti-oxidant enzymes (e.g. SOD), which in turn may lower the accumulation of lipid peroxidation products (MDA levels). 

Nanofibrous mats have been extensively studied as drug delivery systems with controlled or sustained release dynamics. The gradual release of the bioactive drug in the wound environment maintains appropriate concentrations of the drug for longer periods, and this in turn enhances wound repair especially in chronic wounds. We postulate that the beneficial effect we observed for PVA/NAC electrospun mats on wound healing and oxidative stress parameters may be associated in part with their drug release profile. Our NAC-loaded mats showed a biphasic release pattern with a total duration of up to 120 hrs. Most of the drug (approximately 80%) was released from the mats within the first 24 hrs, followed by sustained gradual release thereafter. This release profile is superior to the sudden-burst release pattern, and can be considered acceptable for the *once-daily* topical regimen as tested in the present study. PVA-based electrospun mats are highly vulnerable to burst release of the loaded drug due to the high hydrophilicity of PVA in aqueous media (41). An efficient approach to overcome this disadvantage is to cross-link the nanofibrous mat, an approach we tested by using glutaraldehyde. Mats containing NAC 2.5% and 5% showed a similar drug release profile (Figure 5), and this similarity was confirmed by the regression coefficients obtained in the kinetics models. Based on this finding, the initial rapid release of NAC was likely to be governed by a diffusion-controlled system attributable to the drug’s high hydrophilicity (42). The subsequent sustained release phase may be related to gradual swelling of the cross-linked PVA matrix, which would lead to slower NAC release from the polymer matrix (43). In addition, the probable hydrogen bonding between NAC and PVA molecules (Figure 4) may play part in delaying NAC release into the medium. In similar work, Hou *et al*. fabricated a sandwich-like nanofibrous scaffold with a polycaprolactone nanofiber filling enclosed by layers of collagen/NAC. They demonstrated sustained release of NAC from the mats, and provided *in vitro* and *in vivo* evidence of enhanced wound repair in experimental animals (44). In addition, Zhu *et al*. fabricated a dual-compartment nanofibrous scaffold containing NAC and examined its osteogenic potential in rBMSCs *in vitro*. The release kinetics curve showed that NAC followed a biphasic release profile with an initial burst release followed by a slower, sustained stage. This release profile of NAC was found to maintain high concentrations of the drug, and had a clear effect on the osteogenic differentiation of rBMSCs (34). 

**Table 1:** Explanation of the scale used to evaluate pathology parameters in high-power microscopic fields

Scale	Re-epithelialization	PMNs (#)	Fibroblasts (#)	Collagen density	New vessels(#)	Granulation tissue
0	Absent	Absent	Absent	Absent	Absent	Absent
1	Covering <50% of the wound	<5	1-10	Mild	<5	Mild edematous stroma
2	Covering >50% of the wound	5-15	10-20	Moderate	5-15	Moderate edematous stroma
3	Covering 100% of the wound with keratinization	>15	>20	Abundant	>15	Abundant edematous stroma

**Table 2:** Size, porosity and swelling of PVA-based nanofibers with different NAC concentrations

Fiber composition	Fiber diameter (nm)			Porosity (%) ± sd	Swelling (%) ± sd
	min	max	mean ± sd		
Neat PVA	**186**	**400**	**263 ± ** **62**	78.3 ± 7.0	89.3 ± 7.0
PVA/NAC 2.5%	**181**	**412**	**251 ** **± ** **45**	85.3 ± 6.5	94.6 ± 7.5
PVA/NAC 5%	**153**	**219**	**185** **±** ** 33** ^*#^	89.3 ± 8.5	110.3 ± 8.6

* and # indicate significant difference (*P*<0.05) compared to neat PVA or PVA/NAC 2.5% mats, respectively

NAC: N-acetylcysteine; PVA: Polyvinyl alcohol

PVA: Polyvinyl alcohol

**Table 3:** Regression coefficients obtained from fitting NAC release data to different kinetics models

Mat	R^2^			
	Zero order	First order	Higuchi	Korsmeyer–Peppas (n)
PVA/NAC 2.5%	0.60	0.96	0.95	0.97 (0.46)
PVA/NAC 5%	0.63	0.96	0.96	0.97 (0.54)

n: Diffusion exponent in the Korsmeyer–Peppas model. NAC: N-acetylcysteine, PVA: Polyvinyl alcohol

**Table 4:** Wound area (mean ± standard deviation) on days 3, 7 and 14 post-surgery after topical treatment with normal saline, NAC 5% gel, neat PVA or PVA/NAC 5% mats (n=5–8 per group)

Treatment	Wound area (%)		
	Day 3	Day 7	Day 14
Normal saline	86.7 ± 4.7	56.5 ± 4.7	21.7 ± 4.7
NAC 5% gel	80.1 ± 4.4	49.7 ± 4.2^*^	19.5 ± 4.2
Neat PVA mat	82.3 ± 4.5	53.7 ± 4.5	20.6 ± 4.5
PVA/NAC 5% mat	79.4 ± 6.1	45.2 ± 3.8^*, #^	14.1± 3.0^*, #^

* and # denote statistical significance (*P*<0.05) compared to normal saline or the neat PVA mat, respectively, on each day

NAC: N-acetylcysteine; PVA: Polyvinyl alcohol

* indicates significant differences (P < 0.05) compared to the normal saline group. NAC: N-acetylcysteine, PVA: Polyvinyl alcohol

NAC: N-acetylcysteine; PVA: Polyvinyl alcohol

## Conclusion

The potent anti-oxidant agent NAC was incorporated into PVA solutions to produce electrospun nanofibrous mats characterized by thin and smooth nanofibers with desirable features in terms of porosity, hydrophilicity and *in vitro* drug release profile. The main finding of the present work was that PVA/NAC 5% mats accelerated wound repair efficiently at the macroscopic and microscopic level, and promoted fibroblast proliferation *in vitro*. Biochemical assays also provided evidence for the positive role of anti-oxidant activity of PVA/NAC 5% in wound healing. The main advantages of the fabricated PVA/NAC 5% mats were the presence of NAC as a promising wound healing agent, and a nanofiber scaffold that provided a controlled drug release profile. Although the present findings for fabricated electrospun mats are still far from clinical application in humans, they are a step forward in the design and application of drug-eluting nanofiber scaffolds for wound care.
